# Exploring the predictive power of antinuclear antibodies and Rheumatoid factor correlations in anticipating therapeutic outcomes for female patients with coexisting Sjögren's syndrome and Rheumatoid arthritis

**DOI:** 10.1016/j.jobcr.2025.01.012

**Published:** 2025-02-11

**Authors:** Anitha Krishnan Pandarathodiyil, Hema Shree K, Pratibha Ramani, B. Sivapathasundharam, Ramya Ramadoss

**Affiliations:** a Faculty of Dentistry, SEGi University, Kota Damansara, 47810 Petaling Jaya, Selangor, Malaysia; b Department of Oral and Maxillofacial Pathology, Saveetha Dental College and Hospitals, Saveetha Institute Medical and Technical Science, Saveetha University, Chennai, India; c Department of Oral Pathology, Priyadarshini Dental College, Thiruvallur, Chennai, India; d Department of Oral Biology, Saveetha Dental College and Hospitals, Saveetha Institute Medical and Technical Science, Saveetha University, Chennai, India

**Keywords:** Sjögren's syndrome, Rheumatoid arthritis, Antinuclear antibodies, Rheumatoid factor, Neural network, Treatment response, Autoimmune disease

## Abstract

**Background:**

Sjögren's Syndrome (SS) and Rheumatoid Arthritis (RA) are autoimmune conditions that often coexist in female patients. Biomarkers such as antinuclear antibodies (ANA) and rheumatoid factor (RF) are used for diagnosis, but their predictive power for treatment outcomes remains unclear. This study aims to investigate the correlation between age, ANA, RF, and treatment response in female patients with both SS and RA.

**Objective:**

To evaluate the relationships between age, ANA, RF levels, RA (disease present), and treatment response using Pearson correlation analysis and a neural network model, to predict treatment outcomes in patients with coexisting SS and RA.

**Methods:**

A cohort of 56 female patients aged 30–73 was analyzed. Descriptive statistics provided an overview of key variables, followed by Pearson correlation analysis to assess relationships between age, ANA, RF, RA, and treatment response. A neural network model was developed to predict treatment response based on age, ANA, and RF levels, using a training-to-testing split of 81.3 % and 18.8 %, respectively.

**Results:**

The Pearson correlation analysis revealed a significant positive correlation between age and ANA levels (r = .541, p = 0.031), though no significant correlations were found between age, RF, RA, and treatment response. The neural network model achieved an accuracy of 92.3 % during training and 100 % accuracy during testing for most treatment categories. However, the model struggled to accurately distinguish between certain classes, particularly treatment categories 1 and 3.

**Conclusion:**

Age showed a significant correlation with ANA levels, indicating that older patients may have elevated ANA. The neural network model demonstrated strong predictive power for treatment response, although further refinement is needed to improve its ability to distinguish between all response categories. These findings suggest that machine learning models could enhance personalized treatment strategies for patients with SS and RA, but additional validation with larger datasets is required.

## Graphical abstract

**Figure:**
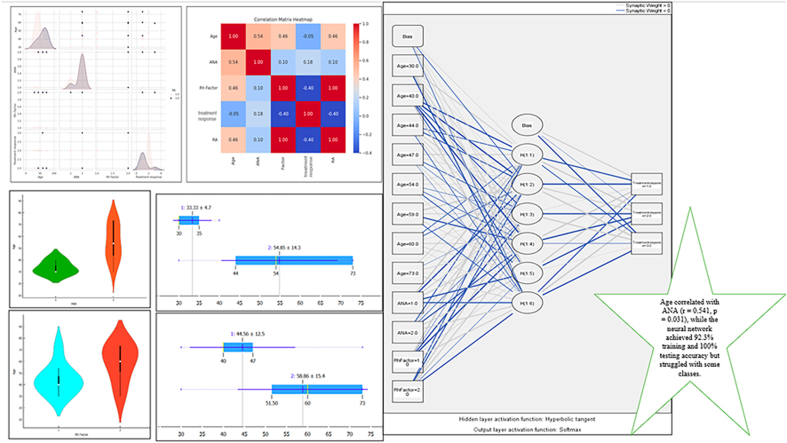

**Key points** **:**

• The study highlights a higher prevalence of Sjögren's Syndrome and Rheumatoid Arthritis in women due to hormonal and genetic factors.
• Examining 56 female patients underscores how these conditions interact and influence treatment outcomes.
• ANA and Rheumatoid Factors are crucial for predicting therapeutic responses.

## 1 Introduction

Sjögren's syndrome (SS), a chronic autoimmune disorder that predominantly affects the exocrine glands and has a significant impact on the quality of life of affected individuals, particularly women.^1^^,^^2^ Sjögren's syndrome is a complex and often misunderstood autoimmune disorder characterized by its preference for exocrine glands, particularly the lacrimal and salivary glands.^2^ This chronic inflammatory condition results in dryness of the mouth and eyes, significantly impacting an individual's quality of life.^3^ While Sjögren's syndrome can affect individuals of any age, gender, or ethnicity, it exhibits a noticeable gender bias, disproportionately affecting women with a female-to-male ratio estimated to be as high as 9:1. This gender disparity highlights the intricate interplay of genetic, hormonal, and environmental factors in the development of this enigmatic disease.^4^

The co-occurrence of SS and Rheumatoid arthritis (RA) presents a considerable challenge in the realm of autoimmune diseases. These persistent inflammatory conditions, which disproportionately affect women, frequently exhibit overlapping symptoms, complex immune dysregulation, and a heightened disease burden. SS primarily affects exocrine glands, resulting in dryness of the mouth and eyes, while RA chiefly impacts synovial joints, causing pain, stiffness, and progressive joint damage.^5^ Despite their distinct clinical presentations, these conditions are linked by the presence of antinuclear antibodies (ANA) and rheumatoid factor (RF).^6^

Identifying ANA and RF plays a crucial role in diagnosing both SS and RA. However, their significance extends beyond diagnosis; they also have the potential to serve as prognostic biomarkers for predicting disease progression and treatment response. This potential is particularly notable in the context of coexisting SS and RA, where the interaction of these autoantibodies may uniquely influence disease severity and treatment outcomes.^7^ Current clinical practice often relies on a trial-and-error approach to managing these conditions, especially when they occur together. While sometimes unavoidable, this approach can lead to delays in achieving optimal therapeutic responses and expose patients to potentially unnecessary side effects. The ability to predict which patients are more likely to respond favorably to specific treatments would represent a significant advancement in personalized medicine for this patient population.^8^

The exploration of ANA and RF correlations as predictive tools is of great importance. Recent findings suggest that specific ANA and RF profiles, including antibody titers, subtypes, and reactivity patterns, may be associated with treatment response and disease activity in both SS and RA. For instance, certain ANA subtypes, such as anti-Ro/SSA and anti-La/SSB, have been linked to specific clinical manifestations and disease severity in both conditions, although with varying implications.^9^ Likewise, RF levels have been correlated with disease activity and radiographic progression in RA. However, the majority of current research primarily focuses on ANA and RF profiles within the context of each disease separately. The combined predictive value of these autoantibodies in patients coping with the dual challenges of SS and RA is largely unexplored.^10^

This research aims to comprehensively investigate the predictive effectiveness of ANA and RF correlations in forecasting treatment outcomes for female patients diagnosed with both SS and RA. Early identification of patients at risk for aggressive disease or poor treatment response could enable timely intervention with more aggressive therapies, potentially slowing disease progression and improving long-term outcomes.

## 2 Materials and methods

### 2.1 Study design

The study encompassed a cohort of 56 female patients, aged between 30 and 73, who had been diagnosed with both SS and RA. The study obtained approval from the Institutional Review Board and secured informed consent from all participants to uphold ethical guidelines and patient confidentiality. Data analysis utilized descriptive statistics, correlation analyses, and neural network modeling to examine the relationships between variables such as age, ANA levels, RF levels, RA presence, and treatment response.

### 2.2 Sample size justification

To ensure adequate statistical power for detecting significant correlations amongst key clinical variables such as ANA levels, Rheumatoid Factor (RF), RA presence, and treatment outcomes, a power analysis was conducted using GPower software. This analysis, based on a medium effect size (r = .3) for correlational studies, an alpha (α) level of .05 (two-tailed) to minimize the risk of Type I errors, and a power level of .80, revealed that a minimum sample size of 50 patients was necessary with three predictors (age, ANA levels, RF levels) included. By utilizing a sample size of 56 patients, the study met the minimum threshold for robust statistical analysis. It is important to acknowledge the need for further validation in larger cohorts.

### 2.3 Patient selection

Female patients who were diagnosed with both SS and RA were identified from clinical databases for recruitment. The inclusion criteria specified patients aged between 20 and 80 years with documented ANA and RF levels, as well as recorded treatment outcomes. One patient was excluded due to incomplete data, resulting in a final sample of 56 patients. The sample size was calculated utilizing G power.

### 2.4 Data collection

Key clinical data were collected from patient records, including age, which was recorded at the time of study enrollment, and ANA levels, measured using standard immunofluorescence assays. Rheumatoid Factor (RF) levels were determined through enzyme-linked immunosorbent assays (ELISA), while the presence of RA was established based on clinical diagnosis. Treatment response was categorized into three classes,^1^^,^^2^^, and 3^ reflecting the degree of symptom improvement and clinical outcomes as reported by healthcare providers.

### 2.5 Descriptive and correlational analysis

Descriptive statistics were used to summarize the dataset, providing a distribution of patient age, ANA levels, RF levels, and treatment responses. Pearson correlation coefficients were calculated to assess relationships between the variables, with significance levels set at p < 0.05 (two-tailed). These correlations provided insights into how clinical markers might relate to disease characteristics and treatment outcomes.

### 2.6 Neural network model

A neural network model was developed to predict treatment responses based on age, ANA levels, and RF levels. While the study cohort included 56 patients with complete and valid data, the neural network model was developed using a subset of 16 patients (13 for training and 3 for testing). This approach was adopted to optimize the model architecture and ensure balanced training and testing phases. The smaller dataset allowed for iterative refinement and validation of the model before scaling up to the full cohort. The model architecture included an input layer with 12 units representing the three input variables (age, ANA levels, and RF levels), along with bias units. The network featured a hidden layer with 6 units and a hyperbolic tangent activation function to introduce non-linearity. The output layer had three units corresponding to the treatment response classes, utilizing a softmax activation function to generate probability distributions for each class. The dataset was divided into training (81.3 %, 13 patients) and testing (18.8 %, 3 patients) subsets. The model was trained using a cross-entropy error function, with early stopping after one consecutive step without improvement in error. During training, the model achieved a cross-entropy error of 1.391 and a 7.7 % incorrect prediction rate, indicating reasonable accuracy. In the testing phase, the model demonstrated improved performance, achieving a cross-entropy error of 4.872E-5 and a .0 % incorrect prediction rate. A confusion matrix was generated to analyze the prediction accuracy.

### 2.7 Justification for neural network selection

A neural network was chosen for this analysis due to its robust capability to model complex, non-linear relationships and perform multi-class classification tasks with high efficacy. The primary advantage of using a neural network lies in its flexibility to capture intricate interactions and dependencies between clinical markers—such as age, ANA levels, and RF levels—that may not be adequately addressed by linear or logistic regression models. Given the study's goal to classify treatment responses into three distinct categories, a neural network's ability to handle non-linearities and interactions provides a significant benefit over more traditional statistical methods.

Moreover, the small sample size of 16 patients presents challenges for statistical models that assume linear relationships and require large datasets to produce stable and reliable estimates. Neural networks, with their capacity to learn from complex patterns and generalize effectively, were deemed particularly suitable for this context. By employing a neural network, the study could leverage its strength in capturing and modeling non-linear relationships and interactions between variables, thereby improving the accuracy and robustness of the treatment response predictions. This approach offers a detailed analysis of the data, enhancing the overall interpretability and reliability of the findings.

### 2.8 Statistical software

All analyses, including descriptive statistics, correlation assessments, and neural network modeling, were conducted using SPSS Neural Networks version 27.0. This software was selected due to its advanced capabilities in handling neural network modeling and its robust suite of statistical tools. SPSS Neural Networks offers a comprehensive platform for developing and training neural network models, allowing for the intricate analysis required in this study. Its user-friendly interface, combined with powerful computational features, enables precise modeling of complex, non-linear relationships and multi-class classifications. In addition to its neural network capabilities, SPSS Neural Networks is well-suited for managing and analyzing clinical datasets.

## 3 Results

### 3.1 Descriptive statistics

The descriptive statistics (Table 1) provide an overview of the distribution of key variables in a sample of 56 female patients with Sjögren's Syndrome, including age, ANA levels, Rheumatoid factor, RA presence, and treatment response. The patients' ages range from 30 to 73 years, with a consistent mean age of 50.81 years and a standard deviation of 16.03, reflecting a diverse age distribution. ANA levels, ranging from 1.0 to 2.0, have a mean of 1.81 (SD = .40), indicating a strong prevalence of positive ANA results within this larger cohort. Rheumatoid factor levels, with a mean of 1.44 (SD = .51), continue to suggest that a substantial proportion of the patients exhibit elevated levels, highlighting the significance of this marker in the clinical profile of these patients. Similarly, the RA variable shows a mean of 1.44 (SD = .51), confirming the association between RA and Sjögren's Syndrome in nearly half of the patients.

**Table 1:** Descriptive statistics of the cohort.

Descriptive Statistics					
	N	Minimum	Maximum	Mean	Std. Deviation
Age	56	30.0	73.0	50.813	16.0342
ANA	56	1.0	2.0	1.812	.4031
Rh Factor	56	1.0	2.0	1.438	.5123
RA	56	1.0	2.0	1.438	.5123
Treatment response	56	1.0	3.0	1.562	.6292
Valid N (listwise)	56				

### 3.2 Pearson correlation between age, ANA, Rh factor, RA, and treatment response

**Fig. 1:**
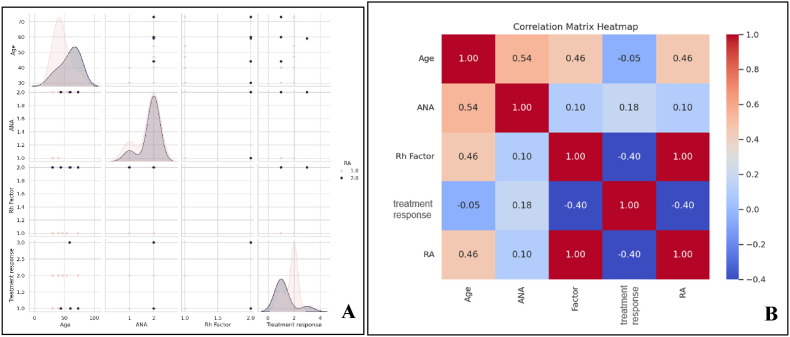
A) Pair plot showing a correlation between age, ANA, RF, RA, and treatment response; 1B) Heat map showing correlation matrix.

The Pearson correlation provides insight into the relationships between age, ANA, RF, RA, and treatment response for the 56 patients in the study (Table 2 and Fig. 1A and B). The scatterplot matrix revealed distinct patterns for continuous and categorical variables. While continuous variables such as age and ANA levels displayed smooth density curves, the "Rh Factor" column lacked a curve due to its categorical nature. As a discrete variable with limited unique values, the distribution of "Rh Factor" could not be represented as a smooth kernel density plot, highlighting the distinction between continuous and categorical data visualization. This observation underscores the importance of considering data type when interpreting distribution patterns in multivariate analyses.

**Table 2:** Pearson correlation between age, ANA, Rh factor, RA, and treatment response.

Correlations						
		Age	ANA	Rh Factor	RA	Treatment response
Age	Pearson Correlation	1	.541∗	.457	.457	−.055
	Sig. (2-tailed)		.031	.075	.075	.840
	N	56	56	56	56	56
ANA	Pearson Correlation	.541∗	1	.101	.101	.181
	Sig. (2-tailed)	.031		.710	.710	.503
	N	56	56	56	56	56
Rh Factor	Pearson Correlation	.457	.101	1	1.000∗∗	−.401
	Sig. (2-tailed)	.075	.710		.000	.124
	N	56	56	56	56	56
RA	Pearson Correlation	.457	.101	1.000∗∗	1	−.401
	Sig. (2-tailed)	.075	.710	.000		.124
	N	56	56	56	56	56
Treatment response	Pearson Correlation	−.055	.181	−.401	−.401	1
	Sig. (2-tailed)	.840	.503	.124	.124	
	N	56	56	56	56	56

#### 3.2.1 Correlation between age and ANA levels

**Fig. 2:**
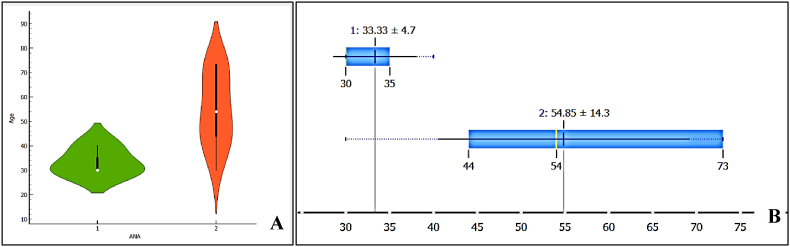
A) shows a scatterplot of age versus ANA levels, with Group 1 (green) representing younger participants and Group 2 (red) representing older participants. The x-axis represents ANA, while the y-axis represents Age. 2B) illustrates the distribution of ANA levels across age groups. Whiskers are omitted to simplify overlapping data. Scores 1 & 2 of ANA represent weaker and stronger positivity based on their dilution. (For interpretation of the references to colour in this figure legend, the reader is referred to the Web version of this article.)

A significant positive correlation was observed between age and ANA levels (r = .541, p = 0.031), as shown in Fig. 2. Panel 2A presents a scatterplot of age versus ANA levels, where Group 1 (green) represents younger participants, and Group 2 (red) represents older participants. The x-axis denotes ANA scores (1 and 2 represent their respective scoring categories), while the y-axis represents age. Panel 2B illustrates the distribution of ANA levels across age groups. Whiskers are omitted in the distribution plot to improve clarity by avoiding overlapping data points. These findings highlight an association between increasing age and elevated ANA levels among the study participants.

#### 3.2.2 Associations of age with rheumatoid factor and RA

**Fig. 3:**
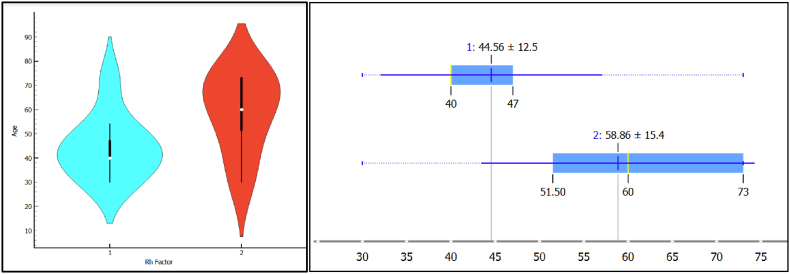
A) Scatter plot showing the correlation between age and RF levels (r = .457, p = 0.075); 3B) Scatter plot showing the correlation between age and RA presence (r = .457, p = 0.075). Group 1 (blue) represents RF-negative patients, while Group 2 (red) represents RF-positive patients. (For interpretation of the references to colour in this figure legend, the reader is referred to the Web version of this article.)

Moderate correlations were noted between age and Rheumatoid Factor (RF) levels (r = .457, *p* = 0.075) as well as between age and RA presence (r = .457, *p* = 0.075), though these did not reach statistical significance (Fig. 3). The violin plots depict the age distribution, with the black bar indicating the interquartile range, the white dot representing the median, and the overall shape illustrating the data density. Whiskers are omitted to focus on the primary data distribution and variability.

#### 3.2.3 Correlation of ANA levels with rheumatoid factor and RA

ANA levels exhibited weak correlations with RF levels (r = .101, *p* = 0.710) and RA presence (r = .101, *p* = 0.710). Both associations were statistically insignificant.

#### 3.2.4 Correlation of age, ANA, rheumatoid factor, and RA with treatment response

**Fig. 4:**
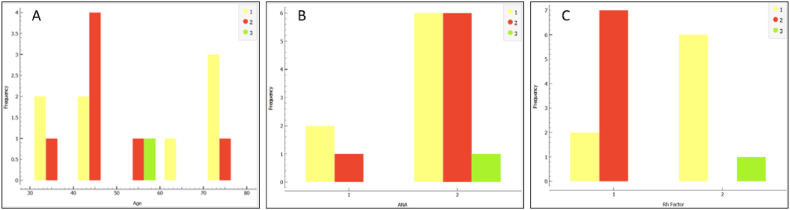
Distribution of treatment responses based on A) Age, B) ANA levels, and C) Rheumatoid factor levels, categorized as 1 - symptomatic relief after Sjögren's treatment, 2 - relief after combined rheumatoid arthritis and 3 - Sjögren's treatment, and no relief after combined rheumatoid arthritis and Sjögren's treatment.

There were no significant correlations found between age (r = −.055, p = 0.840), ANA levels (r = .181, p = 0.503), or RF levels (r = −.401, p = 0.124) and treatment response. However, negative correlations were observed between RF levels and treatment response (r = −.401, p = 0.124) and between RA presence and treatment response (r = −.401, p = 0.124). It's important to note that these associations were not statistically significant (Fig. 4).

### 3.3 Neural network model

The Case Processing Summary (Table 3) details how the data was divided for model training and testing. Among the valid cases, 81.3 % were used for training the model, and the remaining 18.8 % were reserved for testing. This division ensures that the model is trained on most of the data while also being evaluated on a separate set to test its performance. The summary highlights the proportion of data used for each phase of the model's development and assessment.

**Table 3:** Case summary used for testing and training.

Case Processing Summary			
		N	Percent
Sample	Training	13	81.3 %
	Testing	3	18.8 %
Valid		55	100.0 %
Excluded		1	
Total		56	

This neural network is designed to predict treatment response based on three input factors: age, ANA (Antinuclear Antibodies), and Rh Factor. The input layer, consisting of 12 units, processes these factors before passing them to a single hidden layer containing 6 units. The hidden layer employs a hyperbolic tangent activation function to introduce non-linearity, enabling the model to capture complex relationships within the data. With 3 units, the output layer classifies the treatment response into one of three possible categories. A softmax activation function is used in the output layer to produce a probability distribution across these categories, and the model is trained using a cross-entropy error function, which is well-suited for multi-class classification tasks.

**Fig. 5:**
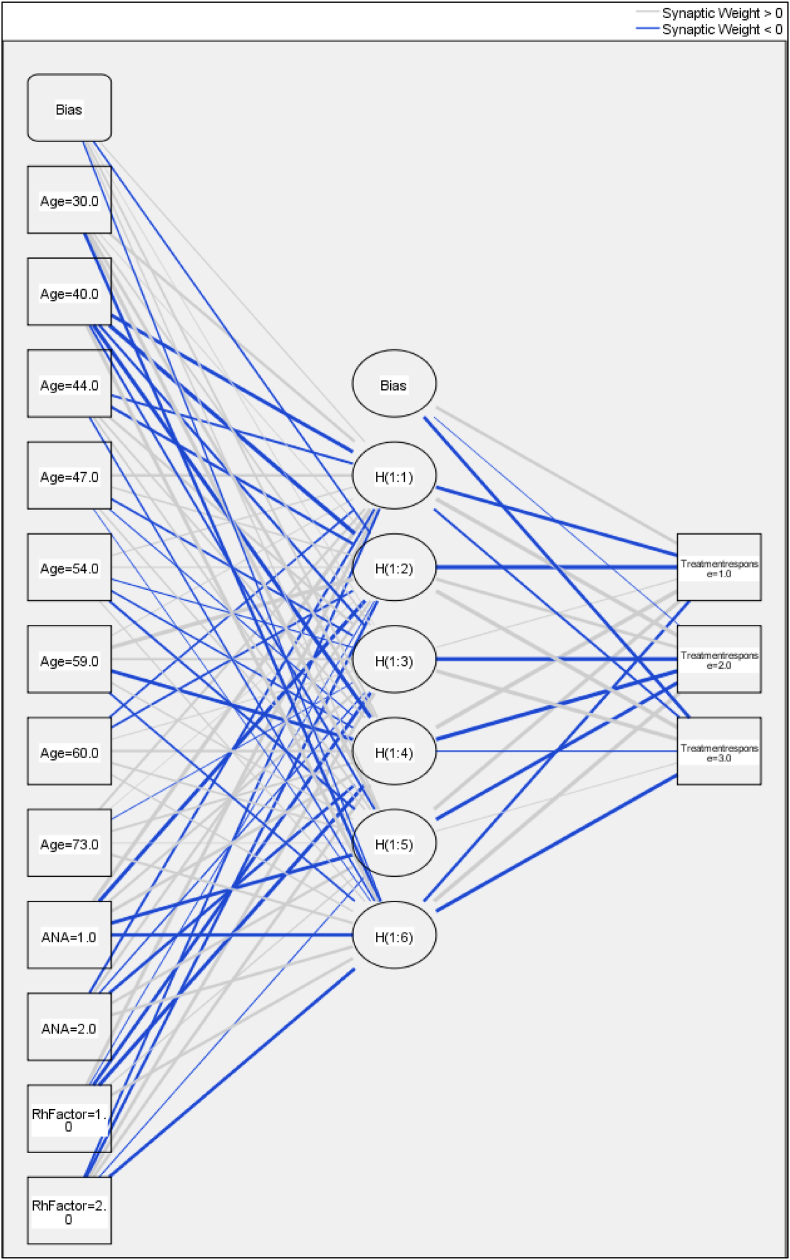
Neural network to predict treatment responses.

The neural network diagram illustrates a model designed to predict treatment responses based on inputs such as age, ANA (Antinuclear Antibodies), and Rh Factor (Fig. 5). The input layer comprises these factors, each represented by various values, and includes a bias unit to adjust the model's performance. These inputs are connected to a hidden layer with six neurons, where the hyperbolic tangent activation function is applied. The connections between the layers are represented by synaptic weights, with gray lines indicating positive weights and blue lines indicating negative weights. The hidden layer outputs are then fed into the output layer, which consists of three units corresponding to different treatment response categories. A softmax activation function in the output layer generates probabilities for each category, enabling the model to classify the input data into one of the three possible treatment responses. The diagram effectively showcases the network's structure, including the bias units and the influence of the input features on the final prediction.

The model indicates that during training, the neural network achieved a cross-entropy error of 1.391, with 7.7 % of predictions being incorrect, suggesting that while the model was reasonably accurate, there was some room for improvement. The training process was efficient, stopping after just one consecutive step without a decrease in error, indicating that the model had likely converged. The training time was recorded as virtually instantaneous, hinting at a small dataset or highly optimized model. The model performed exceptionally well in the testing phase, with a cross-entropy error as low as 4.872E-5 and a .0 % incorrect prediction rate, which accurately predicted the treatment response for every test sample. The dependent variable, treatment response, was predicted with near-perfect accuracy, demonstrating that the model generalizes effectively to new data.

The classification summary reveals that the model performed well during both the training and testing phases in predicting treatment responses across three categories (1.0, 2.0, and 3.0) (Table 4). In the training phase, the model achieved an overall accuracy of 92.3 %, correctly predicting all instances of categories 1.0 and 3.0 with 100.0 % accuracy, though it slightly underperformed with category 2.0, where it misclassified one instance, resulting in an 83.3 % accuracy for that category. During testing, the model demonstrated perfect accuracy, correctly classifying all instances of categories 1.0 and 2.0 with 100.0 % accuracy, while there were no instances of category 3.0 in the test data. Overall, the model's strong performance, especially during testing, indicates its high effectiveness in predicting treatment responses.

**Table 4:** Neural network with accuracy percentage.

Classification					
Sample	Observed	Predicted			
		1.0	2.0	3.0	Percent Correct
Training	1.0	6	0	0	100.0 %
	2.0	1	5	0	83.3 %
	3.0	0	0	1	100.0 %
	Overall Percent	53.8 %	38.5 %	7.7 %	92.3 %
Testing	1.0	2	0	0	100.0 %
	2.0	0	1	0	100.0 %
	3.0	0	0	0	.0 %
	Overall Percent	66.7 %	33.3 %	.0 %	100.0 %

Dependent Variable: Treatment response.

#### 3.3.1 Confusion matrix

**Fig. 6:**
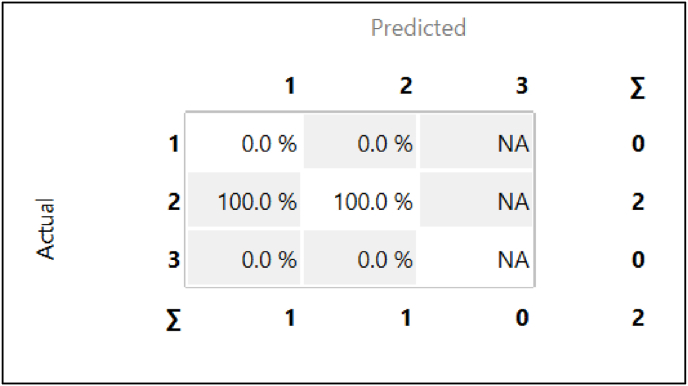
Confusion matrix of classification model.

The confusion matrix (Fig. 6) provides an overview of the classification model's performance across three treatment classes: 1, 2, and 3. The matrix indicates that the model was highly accurate in predicting Treatment Class 2, with 100 % of the instances correctly classified. Both samples belonging to Class 2 were accurately identified by the model. However, the model failed to correctly predict any instances for Treatment Classes 1 and 3, resulting in a .0 % accuracy for both. This suggests that while the model performs well for Class 2, it struggles with distinguishing between Classes 1 and 3.

## 4 Discussion

Managing autoimmune diseases, such as Sjögren's syndrome (SS) and rheumatoid arthritis (RA), involves determining patient treatment responses based on biomarkers and demographic variables, which is of significant interest.^11^ Autoantibodies, specifically antinuclear antibodies (ANA) and Rheumatoid Factor (RF), are pivotal in diagnosing and managing these conditions.^12^ Despite this crucial role, the identification of patients who will respond optimally to treatment remains a complex challenge. This study employs statistical analysis and machine learning techniques to investigate the correlation between age, ANA, RF, RA, and treatment response in female patients diagnosed with SS and RA. In addition to traditional Pearson correlation analysis, a neural network model was developed to predict treatment outcomes based on patient characteristics. This integrated approach not only offers insights into the relationships among key clinical markers and patient demographics but also highlights the potential utility of machine learning models in clinical decision-making.

The descriptive statistics provided a foundational understanding of our cohort of 56 female patients diagnosed with either SS or RA. The wide age range observed (mean = 50.81 years, SD = 16.03) highlights the heterogeneity of this patient population and suggests that age may be a crucial factor influencing the variability in treatment responses. This variability underscores the need for personalized treatment approaches that consider individual patient characteristics, including age.

As expected, the analysis revealed a high prevalence of positive ANA results within the cohort (mean = 1.81, SD = .40), aligning with the established immunological profile of both SS and RA.^13^ Similarly, the mean RF level of 1.44 (SD = .51) further supports the diagnostic relevance of RF as a key marker for these conditions.^14^

A concerning observation is the relatively low mean treatment response score (mean = 1.56, SD = .63), indicating that a significant portion of patients experience limited benefit from current treatment protocols.^15^ This finding emphasizes the urgent need for improved therapeutic strategies for both SS and RA. Future research should focus on identifying novel therapeutic targets and developing personalized treatment plans that optimize patient outcomes.^16^

Of particular interest is the statistically significant positive correlation identified between age and ANA levels (r = .541, p = 0.031). This finding suggests that ANA levels tend to increase with age in this patient population. This observation aligns with previous research indicating that ANA prevalence rises with age, particularly in the context of autoimmune diseases like SS and RA.^17^ This age-related increase in ANA levels could be attributed to the progressive dysregulation of the immune system that occurs with aging.^17^

This observed correlation between age and ANA levels carries significant clinical implications. Firstly, it highlights the importance of considering patient age when interpreting ANA results, as higher levels may not necessarily indicate active disease in older individuals.^18^ Secondly, this finding suggests a potential need for age-adjusted treatment plans. As the immune system undergoes age-related changes, treatment strategies may need to be tailored to account for the evolving immunological landscape in older patients.^19^

While this study provides valuable insights into the clinical characteristics and treatment responses of female patients with SS and RA, it is not without limitations. The relatively small sample size may limit the generalizability of the findings to larger populations. Additionally, the cross-sectional design precludes any conclusions about causality or the temporal relationship between variables.

Future research should focus on larger, longitudinal studies to confirm these findings and further investigate the complex interplay between age, autoimmunity, and treatment response in SS and RA. Further exploration of potential confounding factors, such as disease duration and comorbid conditions, is also warranted.

While age and ANA levels showed a clear positive correlation, the relationships between age and both RF levels (r = .457, p = 0.075) and RA presence (r = .457, p = 0.075) presented more delicate representations. Although trending towards a positive correlation, these associations did not reach statistical significance. This suggests that while a trend might exist for older patients to exhibit higher RF levels and a greater likelihood of RA diagnosis, further investigation is needed. This lack of definitive correlation could be attributed to the inherent heterogeneity of the cohort, which includes patients with varying disease durations and severities.^20^ It is also important to acknowledge that RF elevation is not unique to RA and SS, and its correlation with age may differ across conditions.^21^ Future analyses incorporating additional variables like disease duration or other relevant biomarkers might clarify this relationship.^21^

Interestingly, the analysis revealed weak and statistically insignificant correlations between ANA levels and both RF levels (r = .101, p = 0.710) and RA presence (r = .101, p = 0.710). This finding, while seemingly counterintuitive given their status as hallmark autoantibodies in autoimmune diseases, underscores the complex and often independent behavior of these markers. Several factors could contribute to this observation.^22^ Firstly, ANA and RF levels can fluctuate independently depending on the specific disease state and individual patient characteristics.^23^ Secondly, ANA is often associated with a broader spectrum of systemic autoimmune diseases, while RF demonstrates a more specific link to RA.^24^ This difference in their targeted disease profiles might explain the weak correlation. Finally, the lack of a strong correlation might simply indicate that while both markers are elevated in autoimmune conditions, they are not directly related in a manner that allows for predictable correlations.^25^

Examining the relationship between the studied variables and treatment response revealed no statistically significant correlations with age (r = −.055, p = 0.840), ANA levels (r = .181, p = 0.503), or RF levels (r = −.401, p = 0.124). However, a noteworthy trend emerged with RF levels and RA presence, both showing a negative correlation with treatment response (r = −.401, p = 0.124). While not statistically significant, this negative correlation suggests a potential link between higher RF levels or RA presence and poorer treatment outcomes. This observation aligns with existing literature suggesting that patients with more severe or advanced RA, often characterized by elevated RF levels, tend to experience less favorable treatment responses.^26^ The lack of statistical significance in our findings could be attributed to the limited sample size or the multifaceted nature of treatment response in autoimmune diseases, which is influenced by a complex interplay of factors beyond RF levels or RA diagnosis alone.

The development of a neural network model to predict treatment response based on age, ANA levels, and RF levels represents a novel and promising approach. The model's impressive accuracy rates, particularly during testing, highlight its potential as a valuable tool for clinicians. By inputting these readily available patient characteristics, the model could assist in predicting treatment response and tailoring therapeutic strategies for individual patients.^27^ However, further validation of the model's performance in larger and more diverse patient populations is crucial before widespread clinical implementation.

The neural network model, designed to predict treatment response based on age, ANA levels, and RF levels, demonstrated promising results. The model summary reveals a low training cross-entropy error of 1.391 and an incorrect prediction rate of 7.7 %, indicating effective learning of the relationships between input variables and treatment response. Notably, the model achieved perfect accuracy during testing, with a cross-entropy error of 4.872E-5 and 0 % incorrect predictions. This suggests excellent generalization to unseen data, highlighting its potential as a robust predictive tool.

However, it is crucial to acknowledge the limitations of these findings. The near-perfect accuracy during testing, while encouraging, raises concerns about potential overfitting due to the small sample size. Overfitting occurs when a model learns the training data too well, capturing noise and outliers that hinder its ability to generalize to new data.^28^ To mitigate this risk and validate the model's true performance, future studies should prioritize testing with larger, more diverse datasets and employing cross-validation techniques like k-fold validation.^29^

The model's architecture, featuring an input layer (12 units), a hidden layer (6 units), and an output layer (3 units), is intentionally simplistic. This streamlined design likely contributes to its efficiency and rapid convergence during training. The choice of a hyperbolic tangent activation function in the hidden layer introduces non-linearity, enabling the model to capture complex relationships between age, ANA, and RF levels.^30^ Meanwhile, the softmax activation function in the output layer ensures probabilistic prediction of treatment response, ideal for multi-class classification tasks.^31^

Despite the overall high accuracy, the classification summary reveals a slightly lower accuracy rate of 83.3 % for treatment category 2.0 during training. This suggests that this category might represent a more heterogeneous group regarding treatment response, posing a greater challenge for the model to differentiate between patients. The confusion matrix further supports this observation, highlighting the model's difficulty in accurately classifying instances of Treatment Classes 1 and 3, achieving 0 % accuracy in these cases. This underscores the need for further refinement to improve the model's ability to distinguish between specific treatment categories.

To enhance the model's predictive power and address the limitations highlighted above, several steps are recommended such as incorporating a larger and more diverse cohort would provide a more robust foundation for training and testing, reducing the risk of overfitting and improving generalizability.^32^ Also implementing cross-validation techniques like k-fold validation would provide a more reliable assessment of the model's performance and its ability to generalize to unseen data.^33^ Expanding the feature set beyond age, ANA, and RF levels could significantly enhance the model's predictive accuracy. Including variables like disease duration, other relevant biomarkers (e.g., C-reactive protein, erythrocyte sedimentation rate), and patient-reported outcomes would allow the model to capture a more comprehensive picture of the factors influencing treatment response in autoimmune diseases.^34^^,^^35^

The study presents a novel application of neural network modeling to predict treatment responses in female patients with Sjögren's Syndrome (SS) and Rheumatoid Arthritis (RA), showcasing innovative methodology and clinical relevance. However, its small sample size limits generalizability, with potential overfitting in the neural network and imbalanced performance across treatment categories. Incorporating additional predictors like disease duration and inflammatory markers, alongside cross-validation techniques, could enhance robustness. A longitudinal design would better capture temporal biomarker changes.

By addressing these limitations and incorporating these recommendations, future iterations of this neural network model hold significant promise for advancing personalized medicine in autoimmune disease management. In conclusion, this study presents a comprehensive analysis of the correlations between age, ANA, RF, RA, and treatment response in a cohort of female patients with SS and RA. While traditional Pearson correlations provided insights into the relationships between these variables, the application of a neural network model offers a novel and promising approach to predicting treatment outcomes. However, the results, particularly those from the neural network, require further validation in larger, more diverse cohorts to confirm their clinical utility.

## 5 Conclusion

This study evaluated the predictive relationships between age, ANA, rheumatoid factor, and treatment response in 56 female patients with coexisting Sjögren's Syndrome and Rheumatoid Arthritis, using statistical correlations and a neural network model. Pearson correlation analysis found a significant positive relationship between age and ANA levels, while moderate correlations between age, rheumatoid factor, and RA did not reach significance. Despite no significant correlations with treatment response, the neural network model showed high predictive accuracy, particularly in classifying treatment responses, though it struggled with certain categories, indicating the need for refinement and larger datasets. Overall, the findings highlight the complexity of predicting outcomes in these patients and suggest that data-driven models hold promise for personalized treatment strategies in autoimmune diseases.

## Patient consent

The patient consent was obtained.

## Ethical clearance

Institutional ethical clearance was obtained from IHEC and SRB(scientific review board)

## Sources of funding

There is no funding sources.

## Declaration of competing interest

The authors declare that they have no known competing financial interests or personal relationships that could have appeared to influence the work reported in this paper.
